# Cataract and the increased risk of depression in general population: a 16-year nationwide population-based longitudinal study

**DOI:** 10.1038/s41598-020-70285-7

**Published:** 2020-08-07

**Authors:** Po-Wei Chen, Peter Pin-Sung Liu, Shu-Man Lin, Jen-Hung Wang, Huei-Kai Huang, Ching-Hui Loh

**Affiliations:** 1 School of Medicine, Tzu Chi University, Hualien, Taiwan; 2 Department of Medical Education, Hualien Tzu Chi Hospital, Buddhist Tzu Chi Medical Foundation, Hualien, Taiwan; 3 Center for Aging and Health, Hualien Tzu Chi Hospital, Buddhist Tzu Chi Medical Foundation, No. 707, Sec. 3, Chung Yang Rd., Hualien, 97002 Taiwan; 4 Department of Physical Medicine and Rehabilitation, Hualien Tzu Chi Hospital, Buddhist Tzu Chi Medical Foundation, Hualien, Taiwan; 5 Department of Medical Research, Hualien Tzu Chi Hospital, Buddhist Tzu Chi Medical Foundation, Hualien, Taiwan; 6 Department of Family Medicine, Hualien Tzu Chi Hospital, Buddhist Tzu Chi Medical Foundation, No. 707, Sec. 3, Chung Yang Rd., Hualien, 97002 Taiwan

**Keywords:** Risk factors, Depression, Lens diseases, Vision disorders

## Abstract

Cataract is the primary cause of visual impairment and can be corrected by cataract surgery. We investigated the impact of cataract on the risk of depression along with the benefits of cataract surgery. Patients newly diagnosed with cataract by ophthalmologists between 2001 and 2015 were identified from the National Health Insurance Research Database (NHIRD) in Taiwan. Non-cataract individuals were recruited by 1:1 matching for age, sex and index year. After propensity score matching, 233,258 patients in total were included in our study: 116,629 in each of the cataract and non-cataract cohorts. The primary outcome was the new diagnosis of depression by psychiatrists. In a mean follow-up period of 7.8 years, cataract was significantly associated with increased risk of developing depression (adjusted hazard ratio [aHR] = 1.78, 95% confidence interval [CI] 1.70–1.87, p < 0.001). We further divided the cataract cohort into surgery and non-surgery groups. Notably, cataract surgery group was associated with a decreased risk of depression compared with non-surgery patients (aHR = 0.75, 95% CI 0.71–0.79, p < 0.001). Our results emphasise the importance of regular screening for depression among cataract patients and the beneficial effect of cataract surgery in reducing the risk of depression.

## Introduction

According to the Global Burden of Disease Study, depressive disorders are the third non-fatal leading contributor to the global disease burden in 2017^1^. Depression is associated with functional impairment, risk of dementia, and increased mortality^2^. With insufficient knowledge and stigmatizing attitudes toward depression among Taiwanese people^3^, the situation regarding undiagnosed cases may be worse than that in other countries. In addition, approximately 50% of patients with depression drop out of the treatment before receiving the antidepressant therapy of 6–9 months that is recommended in Taiwan^4^, which will increase the risk of recurrence. Identifying risk factors that can be addressed may help in controlling the burden of depression, both in Taiwan and globally.

In clinical practice, depressive symptoms are common among patients with eye disease, with a pooled prevalence of 25% (range 5.4–57%)^5,6^. However, depression is often left unrecognised or untreated in the ophthalmological clinic, which can negatively affect therapy outcomes and quality of life^5,7^. Previous studies have suggested the correlation between depression and visual impairment^8,9^. A recent nationwide cohort study indicated a longitudinal association between visual impairment and depressive symptoms^10^. Although the leading cause of visual impairment and blindness in the elderly is cataract^11^, studies comparing the risk of depression between cataract patients and healthy individuals are still limited and are either mixed anxiety and depression together^12^, or have selection bias^13^.

Visual impairment caused by cataract is curable with cataract surgery, one of the most common operative procedures performed worldwide because of its high efficacy and minimal complications^14^. However, the impact of cataract surgery on depressive symptoms remains controversial. Some studies have demonstrated the beneficial effects of cataract surgery on depression^15–20^, whereas others have suggested no remarkable impact^13,21,22^.

Depression in cataract patients is an important issue that lacks sufficient evidence. A majority of previous studies are restricted on account of having a cross-sectional design^12,22^, follow-up period less than 3 months^15,18–21^, sample size less than 100^13,15,18,21,22^, and single-institutional bias^15,18–20,22^. In the present study, we performed a nationwide population-based cohort study to examine (1) the long-term association between cataracts and the risk of developing depression and (2) the impact of cataract surgery on the risk of depression.

## Results

### Patient characteristics

Initially, 280,970 participants were included in our study, with 140,485 participants in each of the cataract and non-cataract cohorts following 1:1 matching based on age (in 5-year increments), sex and index year. The overall mean follow-up time was 7.8 years, and the overall mean age was 62.6 years (range 20–101 years). The number of comorbidities was higher in the cataract cohort after age/sex/index year matching. Following 1:1 propensity score matching, there were 116,629 participants in each of the cataract and non-cataract cohorts. All baseline characteristics were well balanced following propensity score matching, with all standardised differences less than 0.1 (Table 1).

**Table 1:** Baseline characteristics of patients with and without cataract.

	Age/sex/index year matching					Propensity score matching				
	Cataract (n = 140,485)		No cataract (n = 140,485)		SMD	Cataract (n = 116,629)		No cataract (n = 116,629)		SMD
	n	%	n	%		n	%	n	%	
**Age (years)**	62.7 ± 10.1		62.5 ± 10.3		0.020	62.9 ± 10.4		62.6 ± 10.5		0.033
< 65	82,315	58.6	83,441	59.4	0.016	66,908	57.4	68,456	58.7	0.027
≥ 65	58,170	41.4	57,044	40.6	0.016	49,721	42.6	48,173	41.3	0.027
**Sex**										
Male	70,556	50.2	70,556	50.2	0.000	57,668	49.5	57,236	49.1	0.007
Female	69,929	49.8	69,929	49.8	0.000	58,961	50.6	59,393	50.9	0.007
**Income (NTD)**										
Dependent	31,673	22.6	32,983	23.5	0.022	27,205	23.3	24,075	20.6	0.065
15,840–29,999	70,049	49.9	71,094	50.6	0.015	56,864	48.8	57,569	49.4	0.012
30,000–44,999	23,851	17.0	22,571	16.1	0.025	19,465	16.7	21,248	18.2	0.040
45,000 or more	14,912	10.6	13,837	9.9	0.025	13,095	11.2	13,737	11.8	0.017
**Comorbidities**										
CCI	1.5 ± 1.9		1.0 ± 1.8		0.291	1.3 ± 1.8		1.2 ± 1.9		0.055
HTN	56,151	40.0	38,951	27.7	0.261	40,589	34.8	38,680	33.2	0.035
DM	35,082	25.0	14,925	10.6	0.382	15,428	13.2	14,925	12.8	0.013
CVA	10,240	7.3	9,150	6.5	0.031	8,705	7.5	8,572	7.4	0.004
Heart failure	3,749	2.7	3,043	2.2	0.033	3,091	2.7	2,870	2.5	0.012
CAD	16,869	12.0	10,296	7.3	0.159	12,460	10.7	10,267	8.8	0.063
Asthma	5,366	3.8	3,692	2.6	0.067	4,358	3.7	3,672	3.2	0.032
COPD	10,111	7.2	7,555	5.4	0.075	8,553	7.3	7,443	6.4	0.038
CKD	3,743	2.7	2,142	1.5	0.080	2,603	2.2	2,132	1.8	0.028
Cirrhosis	1,716	1.2	1,517	1.1	0.013	1,457	1.3	1,406	1.2	0.004
Arthritis	19,076	13.6	11,391	8.1	0.177	13,076	11.2	11,390	9.8	0.047
Malignancy	6,803	4.8	6,641	4.7	0.005	6,057	5.2	6,232	5.3	0.007

Continuous data are expressed as mean ± standard deviation and categorical data are expressed as number and percentage.

*CAD* coronary artery disease, *CCI* Charlson Comorbidity Index, *CKD* chronic kidney disease, *COPD* chronic obstructive pulmonary disease, *NTD* New Taiwan Dollar, *SMD* standardised mean difference.

Within the cataract cohort, 60,454 patients who underwent cataract surgery were classified as the surgery group, and 80,031 patients who did not undergo cataract surgery were classified as the non-surgery group. The median time from first cataract diagnosis to surgery was 398 days. Following 1:1 propensity score matching, there were 58,699 participants in each of the surgery and non-surgery groups, with a balanced distribution of baseline characteristics (Supplementary Table S1).

### Risk of depression in the cataract and non-cataract cohorts

**Figure 1:**
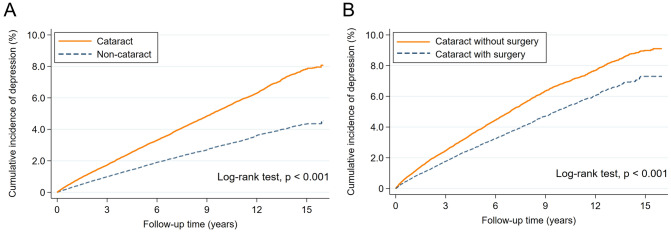
Cumulative incidence of depression in (**a**) cataract and non-cataract cohorts and (**b**) cataract surgery and non-surgery groups in the cataract cohort.

During the mean follow-up period of 7.8 years, Kaplan–Meier analysis revealed that the cumulative incidence of developing depression was consistently higher in the cataract cohort compared with the non-cataract cohort during the entire follow-up period (age/sex/index year matching: 5.43 vs. 2.84 per 1,000 person-years; propensity score matching: 5.37 vs. 3.03; log-rank test, p < 0.001) (Fig. 1A). Diagnosis of cataract was associated with a significantly higher risk of developing depression in univariable (age/sex/index year matching: crude hazard ratio [HR] = 1.92, 95% CI 1.84–2.01, p < 0.001; propensity score matching: crude HR = 1.78, 95% CI 1.70–1.87, p < 0.001) and multivariable (age/sex/index year matching: adjusted HR [aHR] = 1.81, 95% CI 1.73–1.89, p < 0.001; propensity score matching: aHR = 1.72, 95% CI 1.64–1.80, p < 0.001) Cox proportional hazard regression models (Table 2).

**Table 2:** Risk of developing depression in patients with and without cataract.

	Age/sex/index year matching		Propensity score matching	
	Cataract	No cataract	Cataract	No cataract
N	140,485	140,485	116,629	116,629
Events	3,015	6,141	2,620	5,084
Person-years	1,062,304	1,129,970	865,395	945,965
Incidence rate^a^	2.84	5.43	3.03	5.37
**Univariable model**				
HR (95% CI)	1.92 (1.84–2.01)	1 (ref.)	1.78 (1.70–1.87)	1 (ref.)
p value	< 0.001		< 0.001	
**Multivariable model**^b^				
aHR (95% CI)	1.81 (1.73–1.89)	1 (ref.)	1.72 (1.64–1.80)	1 (ref.)
p value	< 0.001		< 0.001	

*CI* confidence interval, *HR* hazard ratio, *aHR* adjusted hazard ratio, *ref*. reference.

^a^Per 1,000 person-years.

^b^Multivariable Cox proportional hazards regression model with adjustments for baseline characteristics shown in Table 1.

Cox regression models revealed a higher risk of developing depression in both the cataract surgery (age/sex/index year matching: aHR = 1.72, 95% CI 1.62–1.82, p < 0.001; propensity score matching: aHR = 1.65, 95% CI 1.54–1.76, p < 0.001) and non-surgery (age/sex/index year matching: aHR = 2.30, 95% CI 2.19–2.41, p < 0.001; propensity score matching: aHR = 2.14, 95% CI 2.03–2.27, p < 0.001) groups compared with the risk of developing depression in the non-cataract cohort (Table 3).

**Table 3:** Risk of developing depression in cataract patients with and without surgery compared with that in non-cataract patients.

	N	Events	Person-years	Incidence rate^a^	Univariable model		Multivariable model^b^	
					HR (95% CI)	p value	aHR (95% CI)	p value
**Age/sex/index year matching**								
No cataract	140,485	3,015	1,062,304	2.84	1 (ref.)		1 (ref.)	
Cataract without surgery	80,031	4,141	588,539	7.04	2.47 (2.35–2.59)	< 0.001	2.30 (2.19–2.41)	< 0.001
No cataract	140,485	3,015	1,062,304	2.84	1 (ref.)		1 (ref.)	
Cataract with surgery	60,454	2,000	381,548	5.24	1.82 (1.72–1.93)	< 0.001	1.72 (1.62–1.82)	< 0.001
**Propensity score matching**								
No cataract	75,381	1,794	550,956	3.26	1 (ref.)		1 (ref.)	
Cataract without surgery	75,381	3,906	555,873	7.03	2.16 (2.04–2.28)	< 0.001	2.14 (2.03–2.27)	< 0.001
No cataract	60,399	1,363	435,722	3.13	1 (ref.)		1 (ref.)	
Cataract with surgery	60,399	1,996	381,237	5.24	1.65 (1.54–1.77)	< 0.001	1.65 (1.54–1.76)	< 0.001

*CI* confidence interval, *HR* hazard ratio, *aHR* adjusted hazard ratio, *ref*. reference.

^a^Per 1,000 person-years.

^b^Multivariable Cox proportional hazards regression model with adjustments for baseline characteristics shown in Table 1.

### Analyses stratified by age and sex

Stratified analyses by age or sex also revealed similar results. A higher risk of depression was found in the cataract cohort compared with the non-cataract cohort in young (< 65 years) and old (≥ 65 years) age groups as well as males and females. Cataract patients with and without surgery had a higher risk of depression compared with the non-cataract controls in all the different sex or age strata. Detailed statistical results were shown in Table 4.

**Table 4:** Age- and sex-stratified analyses for the risk of developing depression according to cataract status and cataract surgery history after propensity score matching.

	Univariable model		Multivariable model^a^	
	HR (95% CI)	p value	aHR (95% CI)	p value
**Age < 65 years**				
No cataract	1 (ref.)		1 (ref.)	
Cataract overall	1.92 (1.81–2.04)	< 0.001	1.82 (1.71–1.94)	< 0.001
Cataract without surgery	2.20 (2.05–2.36)	< 0.001	2.15 (2.01–2.31)	< 0.001
Cataract with surgery	1.80 (1.63–1.99)	< 0.001	1.79 (1.62–1.98)	< 0.001
**Age ≥ 65 years**				
No cataract	1 (ref.)		1 (ref.)	
Cataract overall	1.60 (1.49–1.72)	< 0.001	1.55 (1.44–1.67)	< 0.001
Cataract without surgery	2.09 (1.91–2.29)	< 0.001	2.10 (1.92–2.30)	< 0.001
Cataract with surgery	1.51 (1.37–1.66)	< 0.001	1.50 (1.36–1.65)	< 0.001
**Male**				
No cataract	1 (ref.)		1 (ref.)	
Cataract overall	1.81 (1.68–1.95)	< 0.001	1.69 (1.56–1.82)	< 0.001
Cataract without surgery	2.18 (1.99–2.39)	< 0.001	2.11 (1.93–2.31)	< 0.001
Cataract with surgery	1.64 (1.48–1.82)	< 0.001	1.61 (1.45–1.79)	< 0.001
**Female**				
No cataract	1 (ref.)		1 (ref.)	
Cataract overall	1.78 (1.67–1.89)	< 0.001	1.74 (1.63–1.85)	< 0.001
Cataract without surgery	2.17 (2.02–2.33)	< 0.001	2.16 (2.01–2.32)	< 0.001
Cataract with surgery	1.67 (1.52–1.83)	< 0.001	1.67 (1.52–1.83)	< 0.001

The non-cataract cohort was used as the reference group when calculating HR.

*CI* confidence interval, *HR* hazard ratio, *aHR* adjusted hazard ratio, *ref*. reference.

^a^Multivariable Cox proportional hazards regression model with adjustments for baseline characteristics shown in Table 1.

### Comparison between surgery and non-surgery groups in the cataract cohort

Cumulative incidence curves indicated that the cumulative incidence of developing depression was higher in cataract patients who did not undergo surgery compared with those who did undergo surgery (Fig. 1B). Cox regression models also suggested that cataract patients receiving cataract surgery had a significantly lower risk of depression (age/sex/index year matching: aHR = 0.74, 95% CI 0.70–0.78, p < 0.001; propensity score matching: aHR = 0.75, 95% CI 0.71–0.79, p < 0.001) compared with the risk of depression in those who did not receive surgery (Table 5). Furthermore, the age- and sex-stratified analyses revealed similar findings (Supplementary Table S2).

**Table 5:** Risk of developing depression among cataract patients with and without surgery.

	Age/sex/index year matching		Propensity score matching	
	Cataract surgery	No surgery	Cataract surgery	No surgery
N	60,454	80,031	58,669	58,669
Events	2,000	4,141	1,960	2,996
Person-years	381,548	588,539	369,599	436,290
Incidence rate^a^	5.24	7.04	5.30	6.87
**Univariable model**				
HR (95% CI)	0.73 (0.69–0.77)	1 (ref.)	0.75 (0.71–0.80)	1 (ref.)
p value	< 0.001		< 0.001	
**Multivariable model**^b^				
aHR (95% CI)	0.74 (0.70–0.78)	1 (ref.)	0.75 (0.71–0.79)	1 (ref.)
p value	< 0.001		< 0.001	

*CI* confidence interval, *HR* hazard ratio, *aHR* adjusted hazard ratio, *ref*. reference.

^a^Per 1,000 person-years.

^b^Multivariable Cox proportional hazards regression model with adjustments for baseline characteristics shown in Table 1.

### Discussion

In our nationwide cohort study, we analysed the long-term association between cataract and the risk of depression. Compared with the non-cataract control, the cataract cohort had a higher risk of developing depression (aHR = 1.72) despite adjustment for possible confounders. Furthermore, the aHR increased to 2.14 for cataract patients who did not undergo surgery compared with non-cataract individuals. On the other hand, the risk of depression significantly decreased in cataract patients who underwent surgery (aHR = 0.75) compared with those who did not. These results were consistent regardless of sex and age subgroups.

To date, limited studies have evaluated the differences in the risk of depression between cataract and non-cataract patients^12,13^. McGwin et al. in 2003 found a marginally higher depressive score in cataract patients (7.6 and 5.3 in cataract and non-cataract patients, respectively); this was measured using the Center for Epidemiological Studies-Depression Scale, ranging from 0 to 60^13^. Wang et al.^12^ conducted another community-based survey and found slightly higher odds of mental health contacts for depression or anxiety in cataract patients (OR 1.33). Although these two studies reported a significant but weak association, both had some limitations. The former study enrolled only half of the eligible subjects and had different distributions of age and sex between the cataract and non-cataract patients. The latter study had a cross-sectional design, which cannot represent the longitudinal correlation, and combined depression and anxiety in their outcomes^12^. Therefore, this population-based longitudinal study with a strict diagnosis criterion for depression indicated that cataract is an evident long-term risk factor for depression.

Although some previous studies have evaluated the possible benefit of cataract surgery on depression^13,15–23^, the results are controversial as some of these studies indicated that the depressive symptoms did not improve after cataract surgery^13,21,22^. Moreover, most of these studies had follow-up periods of < 1 year^13,15–23^, and only one was a population based study^16^. Therefore, we conducted this 16-year nationwide cohort study to enhance the reliability and indicated that the risk of depression was reduced by 25% in the cataract surgery group compared with that in the non-surgery group. The result is consistent with that reported by another population-based study that found that the number of mental health contacts for depression or anxiety declined by 18.8% 1 year after cataract surgery^16^.

It is crucial to determine whether further preventive management is required for depression in post-surgical patients. We only found one previous study that included healthy individuals as a control to evaluate the impact of cataract surgery on depressive symptoms^13^. However, that study found an insignificant trend of improvement of depressive symptoms in post-surgical patients compared with non-surgery cataract patients and healthy individuals. It may be due to a relatively small sample size and short follow-up period (342 individuals with up to 1-year follow-up)^13^. The present study also included non-cataract individuals as control. With a larger sample size and long-term follow-up, this study was able to successfully demonstrate a decreased but still higher risk of depression in post-surgical patients compared with healthy individuals. Collectively, these results suggest that while cataract surgery has a beneficial effect in reducing the risk of depression, further attention and preventive management for depression is still required in post-surgical individuals.

Most previous studies have been restricted to older patients with an average age of around 70 years^12,13,16–19,21^ and did not examine whether the correlation between cataract and depression differed between different age sex groups^13,16,19,21^. Therefore, we attempted to include all patients regardless of age and stratified the analyses by age and sex. Increased risk of developing depression was observed in both younger and older groups as well as in males and females. The decreased risk of depression in the surgery group was also noticed in all strata. These results indicate the increased risk of depression in cataract patients and the beneficial effect of cataract surgery in reducing the risk of depression regardless of age and sex.

The exact underlying mechanisms involved in the observed association between cataract and depression remain unclear, but some contributing factors have been hypothesised. Studies have found that visually impaired patients have difficulties in daily activities, especially instrumental activities of daily life and leisure activities^24,25^. Visually impaired individuals rely more on their cognitive function and are consequently more vulnerable to age-related cognitive decline^24^. They also have a higher institutionalisation rate^26^ and visual hallucination risk, which is known as Charles Bonnet syndrome^27,28^. Studies have shown improved quality of life, mental health, and economic state in patients following cataract surgery^15,29^. Therefore, future studies are required to develop a multifactor strategy for the prevention and management of depression in patients with cataract or visual impairment.

From a public health perspective, the present study identified a higher risk of depression in cataract patients. Previous studies have also revealed an increased risk of depression associated with glaucoma^30^ and other eye diseases. The prevalence of depressive symptoms can be as high as 25% for patients with any eye disease^6^. By taking into account all these findings, we suggest that ophthalmologists be vigilant about hidden depression and cooperate with psychiatrists. In addition, as we know that cataract may contribute to the development of depression and that cataract surgery may effectively correct this risk, psychiatrists could ask their patients about their visual acuity condition and refer them to an ophthalmologist if needed.

The strengths of the present study include the nationwide population-based longitudinal design, the strict criteria for cataract and depression that requires diagnosis by specialists, and the direct comparison of cataract patients with non-cataract individuals. However, there are still some limitations that need to be acknowledged here. First, the study design was retrospective and used claims-based data; therefore, we could not obtain granular data on some clinical characteristics such as lifestyle, smoking status and alcohol history. Bias related to unmeasured or unknown confounders was a potential issue given the nature of the study. Second, we used the diagnosis of cataract without obtaining the detailed visual acuity of individuals. This means that the cataract cohort would include more mild cases, which would diminish the impact of cataract on developing depression and steer the results toward the null hypothesis. We were also unable to identify whether patients had cataract in one or both eyes and whether surgery had been performed for both affected eyes. This means that the cataract surgery group included patients who still had an unoperated eye, which may have reduced the observed beneficial effect of cataract surgery. As a result, the association may be stronger than we were able to show. Third, while the strict criteria of depression enhance the diagnostic accuracy, it may result in the underestimated incidence of depression in both the cataract and non-cataract cohorts. Last, the present study was performed in a Taiwanese population; thus, the generalisation of the study findings to populations of other countries, cultures or races remains undetermined, and further studies are required to examine external generalizability.

In summary, the present study is the first nationwide longitudinal study evaluating the impact of cataract and cataract surgery by directly comparing cataract patients with non-cataract individuals. It contributes to the literature by indicating a long-term association of cataract and increased depression risk, demonstrating the beneficial effect of cataract surgery in reducing the risk of depression and clarifying the presence of these correlations regardless of age and sex. The results emphasise the importance of regular screening for depression among cataract patients and the beneficial effect of cataract surgery in preventing depression. Future studies are required to elucidate the underlying mechanisms and develop prevention and management strategies for depression in cataract patients.

## Methods

### Data sources

The present study used data from the Longitudinal Health Insurance Database (LHID) in Taiwan from 2000 to 2016. The LHID contains data from two million enrollees who were randomly sampled from the National Health Insurance Research Database (NHIRD), which includes 99% of the total population in Taiwan. The NHIRD was derived from Taiwan’s National Health Insurance (NHI) programme, which is a single-payer mandatory enrolment healthcare system with universal coverage for outpatient, inpatient, and emergency services. This comprehensive cohort enables the association between cataract and risks of depression to be examined. To protect the privacy and data security, the Health and Welfare Data Science Center encrypted personal identifiers in the LHID. Further details of NHIRD can be found in the previous article^31^. The Research Ethics Committee of Buddhist Tzu Chi General Hospital approved this study protocol (REC No: IRB107-217-B), and the requirement for informed consent was waived due to the use of anonymised data. Our research was performed in accordance with relevant guidelines/regulations.

### Study population

We included a cataract cohort as the exposure group and a non-cataract cohort as the non-exposure (comparison) group. The cataract cohort comprised adult patients diagnosed with cataract between 2001 and 2015 in the LHID. Diagnosis of cataract was defined by at least one inpatient or two outpatient diagnoses by an ophthalmologist, using the International Classification of Diseases, 9th Revision, Clinical Modification (ICD-9-CM diagnosis code 366). The index date was defined as the date of the first diagnosis of cataract. To enhance the likelihood of distinguishing newly diagnosed cataract patients, we excluded patients diagnosed with cataract in 2000. Patients diagnosed with depression (the primary outcome) before cataract diagnosis were also excluded.

The non-cataract cohort was also retrieved from the registry of beneficiaries in the LHID. We applied 1:1 matching based on age (in 5-year increments), sex and index year to identify the non-cataract (comparison) cohort. The index date for paired non-cataract patients was assigned as the same date as that of the matched cataract case. In line with the cataract cohort, patients previously diagnosed with depression before the index date were excluded.

Patients in the cataract cohort were further divided into surgery and non-surgery groups to evaluate the impact of cataract surgery on the risk of depression. Cataract patients who underwent cataract surgery during the follow-up period were identified by order code in the NHIRD and classed as the surgery group. Those who did not undergo surgery were classed as the non-surgery group.

### Outcome measures

The primary outcome was new diagnosis of depression, which required diagnosis by a psychiatrist either once in the inpatient service or twice in the outpatient clinic. The NHIRD used ICD-9-CM codes as diagnostic codes before 2016 then started to follow the ICD-10-CM codes in 2016; therefore, diagnosis of depression was identified by the ICD-9-CM codes from 2000 to 2015 (ICD-9-CM codes 296.2, 296.3, 300.4 and 311) and ICD-10-CM codes in 2016 (ICD-10-CM F32, F33 and F34.1). The follow-up period for each individual was from the index date to the development of depression, death, or December 31, 2016 (the last day in our database). The date of death was identified by linking the LHID to the National Register of Deaths in Taiwan.

### Covariates

We used baseline characteristics from both the outpatient and inpatient reimbursement claims in the LHID as potential confounders. Diagnosis of pre-existing comorbidity was confirmed by at least two clinic diagnoses or one inpatient diagnosis preceding the index date. The Charlson comorbidity index score was calculated to quantify the degree of comorbidities^32,33^. Information on income level was retrieved as a representation of socioeconomic status. The monthly income level was divided into four groups (New Taiwan dollars ≥ 45,000; 30,000–44,999; 15,840–29,999; and financially dependent) based on the income-related NHI premiums as described in detail previously^34^.

### Propensity score matching

To minimise possible selection bias caused by differences in baseline characteristics, we further performed propensity score matching for each direct comparison, including (1) cataract vs. non-cataract cohorts, (2) cataract surgery group vs. non-cataract cohort, (3) cataract non-surgery group vs. non-cataract cohort and (4) surgery vs. non-surgery groups among the cataract cohort. We used a logistic regression model including all baseline covariates listed in Table 1 to calculate a propensity score, which assessed the propensity for belonging to a particular group. Participants in different groups were matched 1:1 based on the nearest-neighbour matching method without replacement, using a calliper width equal to 0.2-times the standard deviation of the logit of the propensity score^35,36^. To ensure that the two different matching methods did not influence our results, all analyses were conducted using both the initial age, sex and index year matching and 1:1 propensity score matching.

### Statistical analyses

We used a *t* test for continuous variables and the χ^2^ test for categorical variables for comparison of baseline characteristics. In the survival analysis, we used Kaplan–Meier methods to estimate the cumulative incidences with log-rank tests for comparison between cumulative incidence curves. Univariable and multivariable Cox proportional hazard regression models were performed to calculate HRs and 95% confidence intervals (CIs) for the risk of developing depression. All baseline characteristics listed in Table 1 (including age, sex, financial state and comorbidities) were considered as covariates and adjusted by multivariable Cox proportional hazard regression models to reduce possible confounding effects. To diminish any potential bias caused by immortal time before surgery in the cataract surgery group, we redefined the index date as the date of surgery instead of the date of diagnosis of cataract when performing comparisons related to the surgery group. We further performed stratified analyses according to age and sex subgroups. Standardised difference was used to assess any differences in baseline characteristics between groups, and a value of < 0.1 was considered negligible^37^. A two-sided probability value < 0.05 was considered statistically significant. All statistical analyses were performed using SAS 9.4 software (SAS Institute, Inc., Cary, NC, USA, RRID: SCR_004635) and Stata version 15 (Stata Corporation, College Station, TX, USA, RRID: SCR_012763).

## Supplementary information

Supplementary file1

## Data Availability

The dataset used in this study is managed by the Taiwan Ministry of Health and Welfare, and, thus, cannot be made available publicly. Health and Welfare Data Science Center, Ministry of Health and Welfare, Taiwan has approved our access to the dataset. Researchers interested in accessing this dataset can submit a formal application to the Ministry of Health and Welfare to request access (https://dep.mohw.gov.tw/DOS/cp-2516-3591-113.html). All data generated or analysed during this study are included in this article and its supplementary information files.
