# Supplementary material for: Cataract and the increased risk of depression in general population: a 16-year nationwide population-based longitudinal study
Source: Sci Rep. 2020 Aug 7;10:13421. doi: 10.1038/s41598-020-70285-7 (PMC7414888; doi:10.1038/s41598-020-70285-7)
Supplement: Supplementary file 1 — Supplementary file1 [file 41598_2020_70285_MOESM1_ESM.docx]

**Cataract and the increased risk of depression in general population: a 16-year nationwide population-based longitudinal study**

Po-Wei Chen^1,2,†^, MD; Peter Pin-Sung Liu^3,†^, MS; Shu-Man Lin^1,4^, MD; Jen-Hung Wang^5^, MS; Huei-Kai Huang^1,5,6,^*, MD; Ching-Hui Loh^1,3,^*, MD, PhD

^1^School of Medicine, Tzu Chi University, Hualien, Taiwan

^2^Department of Medical Education, Hualien Tzu Chi Hospital, Buddhist Tzu Chi Medical Foundation, Hualien, Taiwan

^3^Center for Aging and Health, Hualien Tzu Chi Hospital, Buddhist Tzu Chi Medical Foundation, Hualien, Taiwan

^4^Department of Physical Medicine and Rehabilitation, Hualien Tzu Chi Hospital, Buddhist Tzu Chi Medical Foundation, Hualien, Taiwan

^5^Department of Medical Research, Hualien Tzu Chi Hospital, Buddhist Tzu Chi Medical Foundation, Hualien, Taiwan

^6^Department of Family Medicine, Hualien Tzu Chi Hospital, Buddhist Tzu Chi Medical Foundation, Hualien, Taiwan

^†^ These authors should be considered joint first authors.

* These authors should be considered joint corresponding authors.

**SUPPLEMENTAL INFORMATION**

**Supplementary Table S1.** Baseline characteristics of cataract patients with and without cataract surgery

|  | Cataract cohort | | | | |  | Propensity score matching | | | | |
| --- | --- | --- | --- | --- | --- | --- | --- | --- | --- | --- | --- |
|  | Cataract Surgery  (n = 60,454) | | No surgery  (n = 80,031) | | SD |  | Cataract surgery  (n = 58,699) | | No surgery  (n = 58,699) | | SD |
|  | n | % | n | % |  |  | n | % | n | % |  |
| Age (years) | 64.3 ± 10.5 | | 61.6 ± 9.7 | | 0.267 |  | 63.9 ± 10.4 | | 63.3 ± 9.9 | | 0.062 |
| <65 | 30,041 | 49.7 | 52,274 | 65.3 | 0.320 |  | 29,999 | 51.1 | 32,871 | 56.0 | 0.098 |
| ≥65 | 30,413 | 50.3 | 27,757 | 34.7 | 0.320 |  | 28,700 | 48.9 | 25,828 | 44.0 | 0.098 |
| Sex |  |  |  |  |  |  |  |  |  |  |  |
| Male | 32,287 | 53.4 | 38,269 | 47.8 | 0.112 |  | 31,102 | 53.0 | 32,166 | 54.8 | 0.036 |
| Female | 28,167 | 46.6 | 41,762 | 52.2 | 0.112 |  | 27,597 | 47.0 | 26,533 | 45.2 | 0.036 |
| Income (NTD) |  |  |  |  |  |  |  |  |  |  |  |
| Dependent | 13,610 | 22.5 | 18,063 | 22.6 | 0.001 |  | 13,356 | 22.8 | 13,476 | 23.0 | 0.005 |
| 15,840–29,999 | 32,483 | 53.7 | 37,566 | 46.9 | 0.136 |  | 31,037 | 52.9 | 30,945 | 52.7 | 0.003 |
| 30,000–44,999 | 8,665 | 14.3 | 15,186 | 19.0 | 0.125 |  | 8,644 | 14.7 | 8,522 | 14.5 | 0.006 |
| 45,000 or more | 5,696 | 9.4 | 9,216 | 11.5 | 0.069 |  | 5,662 | 9.7 | 5,756 | 9.8 | 0.005 |
| Comorbidities |  |  |  |  |  |  |  |  |  |  |  |
| CCI | 1.5 ± 1.9 | | 1.5 ± 1.9 | | 0.016 |  | 1.5 ± 1.9 | | 1.5 ± 1.9 | | 0.011 |
| HTN | 25,099 | 41.5 | 31,052 | 38.8 | 0.056 |  | 24,232 | 41.3 | 24,134 | 41.1 | 0.004 |
| DM | 15,006 | 24.8 | 20,076 | 25.1 | 0.006 |  | 14,697 | 25.0 | 14,981 | 25.5 | 0.011 |
| CVA | 4,360 | 7.2 | 5,880 | 7.4 | 0.005 |  | 4,266 | 7.3 | 4,226 | 7.2 | 0.003 |
| Heart failure | 1,867 | 3.1 | 1,882 | 2.4 | 0.046 |  | 1,810 | 3.1 | 1,640 | 2.8 | 0.017 |
| CAD | 7,696 | 12.7 | 9,173 | 11.5 | 0.039 |  | 7,528 | 12.8 | 7,175 | 12.2 | 0.018 |
| Asthma | 2,676 | 4.4 | 2,690 | 3.4 | 0.055 |  | 2,568 | 4.4 | 2,337 | 4.0 | 0.020 |
| COPD | 4,968 | 8.2 | 5,143 | 6.4 | 0.069 |  | 4,773 | 8.1 | 4,430 | 7.6 | 0.022 |
| CKD | 1,823 | 3.0 | 1,920 | 2.4 | 0.038 |  | 1,766 | 3.0 | 1,658 | 2.8 | 0.011 |
| Cirrhosis | 795 | 1.3 | 921 | 1.2 | 0.015 |  | 784 | 1.3 | 736 | 1.3 | 0.008 |
| Arthritis | 8,391 | 13.9 | 10,685 | 13.4 | 0.016 |  | 8,024 | 13.7 | 7,867 | 13.4 | 0.008 |
| Malignancy | 2,703 | 4.5 | 4,100 | 5.1 | 0.030 |  | 2,678 | 4.6 | 2,502 | 4.3 | 0.015 |

Continuous data are expressed as mean ± standard deviation, and categorical data are expressed as number and percentage.

Abbreviations: CAD, coronary artery disease; CCI, Charlson Comorbidity Index; CKD, chronic kidney disease; COPD, chronic obstructive pulmonary disease; NTD, New Taiwan Dollar; SD: standardized difference.

**Supplementary Table S2.** Age- and sex-stratified analyses for the risk of developing depression in cataract patients with and without surgery after propensity score matching

|  | Univariable model | |  | Multivariable model^a^ | |
| --- | --- | --- | --- | --- | --- |
|  | HR (95% CI) | p-value |  | aHR (95% CI) | p-value |
| Age < 65 years |  |  |  |  |  |
| Cataract without surgery | 1 (ref.) |  |  | 1 (ref.) |  |
| Cataract with surgery | 0.80 (0.74–0.86) | <.001 |  | 0.79 (0.73–0.86) | <.001 |
| Age ≥ 65 years |  |  |  |  |  |
| Cataract without surgery | 1 (ref.) |  |  | 1 (ref.) |  |
| Cataract with surgery | 0.71 (0.65–0.77) | <.001 |  | 0.71 (0.65–0.77) | <.001 |
| Male |  |  |  |  |  |
| Cataract without surgery | 1 (ref.) |  |  | 1 (ref.) |  |
| Cataract with surgery | 0.75 (0.69–0.82) | <.001 |  | 0.75 (0.69–0.81) | <.001 |
| Female |  |  |  |  |  |
| Cataract without surgery | 1 (ref.) |  |  | 1 (ref.) |  |
| Cataract with surgery | 0.75 (0.69–0.81) | <.001 |  | 0.75 (0.69–0.81) | <.001 |

Cataract patients without surgery were used as the reference group when calculating HR.

^a^ Multivariable Cox proportional hazards regression model with adjustments for all baseline characteristics shown in Table 1.

Abbreviations: CI, confidence interval; HR, hazard ratio; aHR, adjusted hazard ratio; ref., reference
